# Spatio-Temporal Correlation Tensors Reveal Functional Structure in Human Brain

**DOI:** 10.1371/journal.pone.0082107

**Published:** 2013-12-05

**Authors:** Zhaohua Ding, Allen T. Newton, Ran Xu, Adam W. Anderson, Victoria L. Morgan, John C. Gore

**Affiliations:** 1 Vanderbilt University Institute of Imaging Science, Nashville, Tennessee, United States of America; 2 Department of Radiology and Radiological Sciences, Vanderbilt University, Nashville, Tennessee, United States of America; 3 Department of Biomedical Engineering, Vanderbilt University, Nashville, Tennessee, United States of America; 4 Department of Electrical Engineering and Computer Science, Vanderbilt University, Nashville, Tennessee, United States of America; 5 Chemical and Physical Biology Program, Vanderbilt University, Nashville, Tennessee, United States of America; 6 Monroe Carell Jr. Children's Hospital, Vanderbilt University, Nashville, Tennessee, United States of America; 7 Department of Physics and Astronomy, Vanderbilt University, Nashville, Tennessee, United States of America; 8 Department of Molecular Physiology and Biophysics, Vanderbilt University, Nashville, Tennessee, United States of America; Hangzhou Normal University, China

## Abstract

Resting state functional magnetic resonance imaging (fMRI) has been commonly used to measure functional connectivity between cortical regions, while diffusion tensor imaging (DTI) can be used to characterize structural connectivity of white matter tracts. In principle combining resting state fMRI and DTI data could allow characterization of structure-function relations of distributed neural networks. However, due to differences in the biophysical origins of their signals and in the tissues to which they apply, there has been no direct integration of these techniques to date. We demonstrate that MRI signal variations and power spectra in a resting state are largely comparable between gray matter and white matter, that there are temporal correlations of fMRI signals that persist over long distances within distinct white matter structures, and that neighboring intervoxel correlations of low frequency resting state signals showed distinct anisotropy in many regions. These observations suggest that MRI signal variations from within white matter in a resting state may convey similar information as their corresponding fluctuations of MRI signals in gray matter. We thus derive a local spatio-temporal correlation tensor which captures directional variations of resting-state correlations and which reveals distinct structures in both white and gray matter. This novel concept is illustrated with *in vivo* experiments in a resting state, which demonstrate the potential of the technique for mapping the functional structure of neural networks and for direct integration of structure-function relations in the human brain.

## Introduction

Magnetic resonance imaging (MRI) has permeated many aspects of neuroscience and is widely used for studying the structure and functional architecture of the brain. Two types of imaging in particular, viz. functional MRI (fMRI) based on imaging changes in BOLD (blood oxygenation level dependent) signals, and diffusion tensor imaging (DTI) based on quantification of the anisotropy of water movements in white matter fibers, have contributed enormously to our ability to assess functional activity in cortical areas and the fine structure of white matter tracts respectively [1]–[4]. Collectively these enable the interrogation of how localized volumes in the brain are engaged in specific functions and how separate regions are anatomically connected. In addition, evaluations of the temporal correlations between voxels of low frequency BOLD fluctuations in a resting or steady state are believed to provide direct measurements of the functional connectivity between cortical regions [5], [6], and thereby reveal which distributed neural circuits underlie various brain functions. There is considerable interest and potential importance in extending such MRI methods and in combining their different types of information to achieve a greater understanding of the functional anatomy of the brain [7], [8].

The studies described here were motivated by three considerations. First, although there is considerable motivation and potential applications for combining structural tractography obtained from DTI with functional information obtained by fMRI, at present these different imaging methods provide only complementary information and do not lend themselves to direct integration [9]. Rather, because fMRI signals arise from gray matter, in which water appears to diffuse largely isotropically, and DTI data are derived from white matter, from which task-based activation signals have not been robustly obtainable, most current approaches simply identify separated cortical sites of activation and attempt then to connect them via resting state correlations of BOLD signals and/or white matter tract tracings (e.g., [10]–[14]). There is no overlap in the biophysical origins of these different data sets, so a method for directly fusing fMRI and DTI may potentially provide new abilities to integrate and interpret structure and function.

Second, in general, task-based activations within white matter have not been routinely observed, and the conventional explanation is that white matter blood volume and flow are much lower than in gray matter, and the energy requirements for electrical activity and hemodynamic response in white matter are different. However, more recent studies of task-induced activation report significant BOLD effects within the corpus callosum [15], suggesting that MRI signals related to underlying neuronal activity may be encoded in these regions. Moreover, it is also apparent that fluctuations in resting state BOLD signals need not be directly related to task-based changes, and may be relatively greater when baseline flow is lower. The mean signal intensity in typical BOLD-sensitive acquisitions in gray matter is typically only 5–20% higher than from white matter, and the coefficient of temporal variation in white matter is 60–90% of that in gray matter [16]. We expect that larger *fractional* changes in blood flow or volume may occur to meet increased metabolic demands when the baseline flow is low, as is also seen in gray matter.

Third, examination of the power spectra of MRI signals from white matter in a resting state show significant components at the low frequencies (*f* = 0.01 – 0.1 Hz) associated with evidence of functional connectivity in gray matter. Indeed, even after correcting for global time course variations, the percent power in the range 0.01 to 0.1 Hz within white matter is about 10% of the total variance, comparable to that in gray matter (see the Results Section for more detail). These observations are all consistent with the postulate that resting state variations that reflect neural activity may be observable in white matter as well as gray matter even if task-based activations are not detectable.

Taken together, these considerations motivated our *hypothesis* that appropriate analysis of resting state acquisitions may reveal MRI signal variations within white matter that reflect neural electrical activity and the propagation of information. As such we might also expect to see patterns of BOLD signal variations that exhibit anisotropic correlations with neighboring regions and are associated with major white matter tracts. To evaluate this hypothesis, we examined the correlations of voxels within white matter in resting state acquisitions with their nearest neighbors. For every voxel this creates a matrix of 26 inter-voxel values which may then be used to construct a local spatio-temporal correlation tensor. In gray matter we expect these tensors to be largely isotropic except at the boundaries of functional domains. In white matter, evidence of anisotropy would be consistent with our hypothesis and could form the basis of a new way to integrate directly the structure and function of neural networks in the human brain.

## Methods

### Ethics Statement

The protocol used in this study was approved by Vanderbilt University Institutional Review Board. Prior to experiments, written informed consent was obtained from each participant.

Six healthy volunteers (three females, mean age = 28.5 yrs, age range = 24–34 yrs) participated in this study. For each subject, a set of anatomical and diffusion weighted MRI (DW-MRI) data and resting state BOLD signals were acquired on a 3T MRI scanner. For each subject both spatio-temporal correlation tensors and diffusion tensors were computed. Axonal fibers were tracked from the DW-MRI data and temporal correlations of resting state BOLD signals along the fiber tracts were measured. In addition, the spatial distributions of temporal correlations of resting state signals to seed voxels in white matter were examined for selected regions, and pathways reconstructed from spatio-temporal tensors were mapped. Details for key procedures in this study are described below.

### *In vivo* resting state fMRI and diffusion imaging

All imaging was performed on a 3T Philips Achieva scanner (Best, Netherlands) installed at Vanderbilt University Institute of Imaging Science. Subject motion was minimized by using pads placed within the head coil.

#### Image Acquisitions

Anatomical MRI: T_1_-weighted (T1w) images were obtained using a multi-shot gradient echo (GE) sequence with TR = 8.9 ms, TE = 4.6 ms, matrix size = 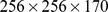, and voxel size = 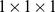
mm^3^. DW-MRI: A single-shot, spin echo (SE), echo-planar imaging (EPI) sequence was used to acquire DW-MRI data with *b* = 1000 s/mm^2^, 32 diffusion-sensitizing directions, TR = 10 s, TE = 60 ms, SENSE factor = 3, matrix size = 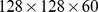, and voxel size = 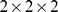
mm^3^. To improve the image signal-to-noise ratio, three repeated scans were acquired. FMRI: Images sensitive to BOLD contrast were acquired using a T_2_*-weighted GE EPI sequence and the following parameters: TR = 2 s, TE = 35 ms, matrix size = 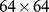, FOV = 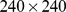 mm^2^, 30 slices of 4.5 mm thickness with a 0.5 mm gap, and 200 dynamics. Subjects were instructed to close eyes without performing any functional tasks.

#### Image Processing

First, the three repeated DW-MRIs were co-registered and averaged, from which diffusion tensor elements were calculated using linear least squares fitting [17]. Second, the resting state fMRI data were corrected for slice timing and head motion using standard spm2 tools (http://www.fil.ion.ucl.ac.uk/spm/software/spm2). Subject data with movement more than 2 mm of translation or 2° of rotation in any direction were excluded. The corrected data were then co-registered with the b = 0 DW-MRI volume. For each subject, a global time course was removed by intensity normalization, i.e., equalizing the mean values of all volumes in the data. The voxels in the brain were low pass filtered with a cutoff frequency of 0.1 Hz. Spatio-temporal correlation tensors were constructed voxel-wise, as described in the sub-section below, using Pearson's linear correlation coefficients (*r*) of BOLD signals and *C* = *r* ^2^.

### Construction of spatio-temporal correlation tensor

The BOLD-sensitive MRI signal from within each voxel provides a time series that exhibits small amplitude fluctuations. The temporal correlation between pairs of voxels indicates their degree of synchronous variation. For any single voxel, a correlation tensor can be constructed to characterize the covariation of neighboring voxels, which is mathematically similar to the construction of diffusion tensors in DTI experiments [17]. Assuming only a first tier neighborhood of 26 voxels is used, a direction vector connecting a voxel of interest and each voxel in the neighborhood is defined, which is subsequently normalized into a unit vector. For a spatio-temporal correlation tensor **T** to be constructed, the estimated correlation *C* projected along a vector **n** *_i_* (*x*_i_, *y*_i_, *z*_i_) is



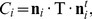
(1)where *t* denotes a transpose operation.

Let **C** denote observed temporal correlations along the 26 directions, **C** = (*C*_1_, *C*_2_, …, *C*_26_)*^t^*. After rearranging the tensor **T** into a column vector **T** *_c_*, the relation between **C** and **T** *_c_* can be expressed as



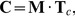
(2)where **M** is a design matrix of size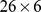. The *i* ^th^ row of **M** has the form of 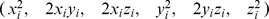
_._ A least squares solution for the column vector **T** *_c_* can be obtained as follows:



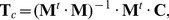
(3)where the superscript *-1* denotes matrix inverse.

Similar to the diffusion tensor, the eigenvector of the spatio-temporal correlation tensor **T** corresponding to the largest eigenvalue (the major eigenvector) represents the dominant direction of temporal correlation.

### Fiber tracking with diffusion tensor images

To examine correlation profiles along anatomic fiber tracts, streamline fiber tracking was performed with DTI data acquired during the same session. For each subject, a tracking process was launched from white matter seed voxels selected by identifying all voxels with FA>0.6. Fibers were traced using a step of 0.05 voxel until either of two conditions was satisfied: reaching a voxel with FA<0.4 or reaching a step-wise change in fiber direction greater than 30°. This identified a set of fiber tracts that were confined to major white matter pathways.

Temporal correlations of resting state BOLD signals were measured between voxel pairs lying on the same fiber tract defined by DTI above, and these correlations were compared to those measured between pairs of voxels randomly sampled in the brain with FA>0.4. This serves to test whether the pair-wise correlations measured along a fiber tract are different from the background correlations measured throughout the brain, and how this changes as a function of the fiber length (separation distance). The number of randomly sampled voxel pairs was chosen to be equal to the number of on-fiber voxel pairs at each given separation. The mean correlation amongst all pairs of voxels in both the on-fiber or random samples was compared, and t-tests were performed to test for significant differences.

### Probabilistic tracking of optic radiation pathways

To demonstrate the potential of spatio-temporal correlation tensors for visualizing long range functional structures, pathways of the left optic radiation were reconstructed using probabilistic tracking from the maps of correlation tensors derived. First, a seed region of
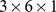
cubic voxels was defined. Similar to [18], the location was chosen to be distal to the Meyer's Loop, so as to avoid difficulties in following rapid directional changes around the loop. Second, we defined the target region to be the primary visual cortex (Brodmann Area 17), using the WFU PickAtlas tool [19]. The probabilistic tracking process began with random sampling of the 18 voxels in the seed region. For each sampled seed voxel, a pathway was reconstructed sequentially at a step size of 0.5 voxel until it reached the target region or a step-wise change in path direction was greater than 60°. At each step, the path followed the major eigen direction of a tensor that was randomly sampled from an average of the incoming tensor and the current tensor.

The probabilistic tracking process was repeated 100,000 times, and the pathways that reached the target region were retained. From these pathways, maps of probability density were computed to record the ratio of the number of times that each voxel was traversed to the total number of pathways. Maps of mean path direction were also computed voxel-wise to record the mean direction of all paths traversing the voxel. With these two maps, streamline tracking was implemented from the target region (left primary visual cortex) with cut-off probability density of 0.01, in which the direction opposite to that of mean path direction was followed sequentially. The backward tracking process was terminated when it reached the seed region.

## Results

### Intensity profiles and power spectra of MRI signals in gray and white matter

**Figure 1:**
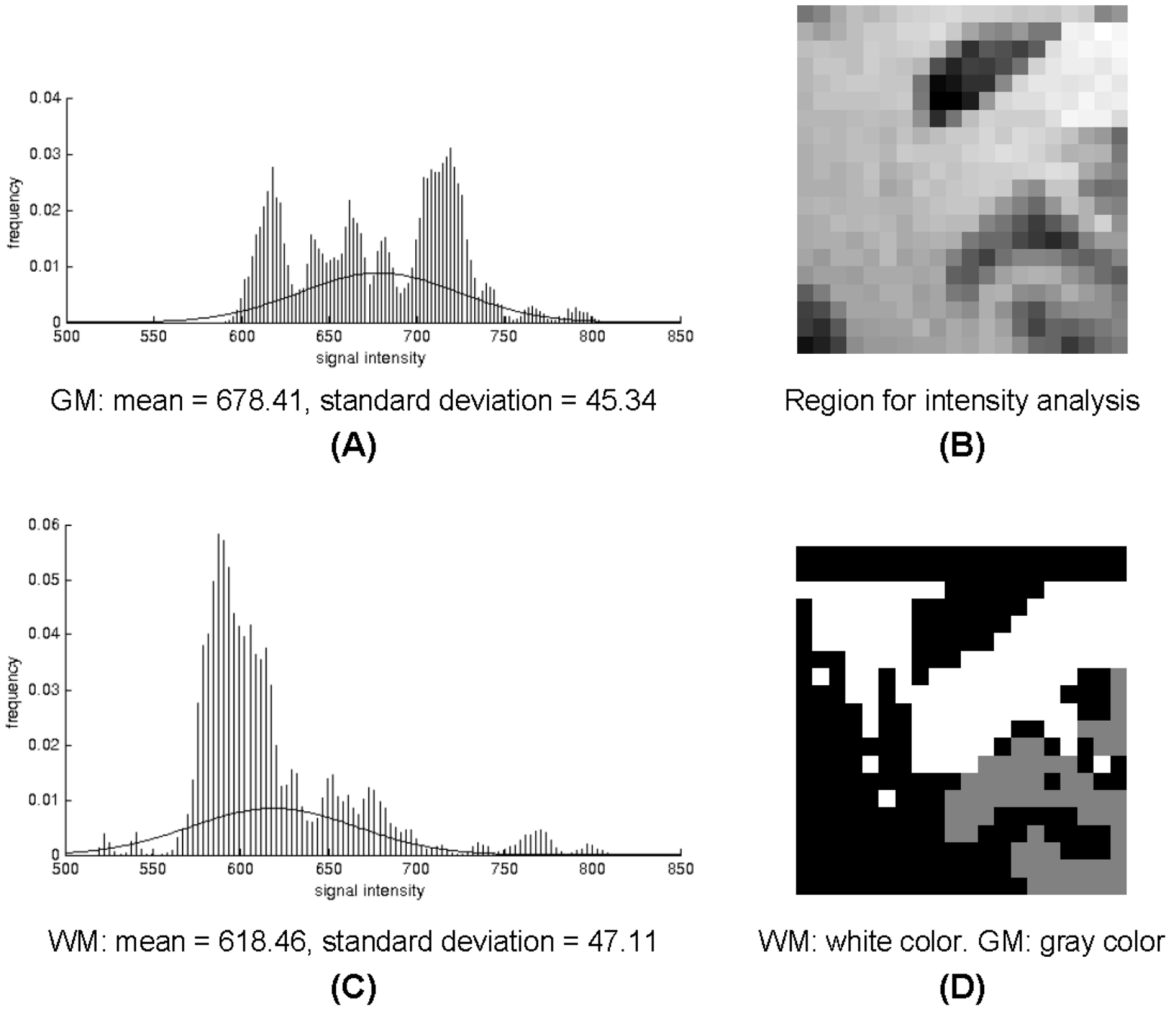
Intensity profiles of BOLD signals in GM (A) and WM (C). Image in (B) is an enlarged view of the boxed region in Fig. 3D, which is segmented into WM and GM using intensity thresholding (D). Cerebro-spinal fluid and regions of WM-GM intensity overlapping are denoted in black and excluded from analysis.

Histograms of BOLD-sensitive MRI signal intensities from gray matter (GM) and white matter (WM) for a selected region are shown in the left column of Fig. 1. Mean signal intensity in WM is ∼91% of that in the GM, and their standard deviations are comparable (≈7%). Voxel-averaged temporal variations of MRI signals in the WM are ∼82% that in the GM.

**Figure 2:**
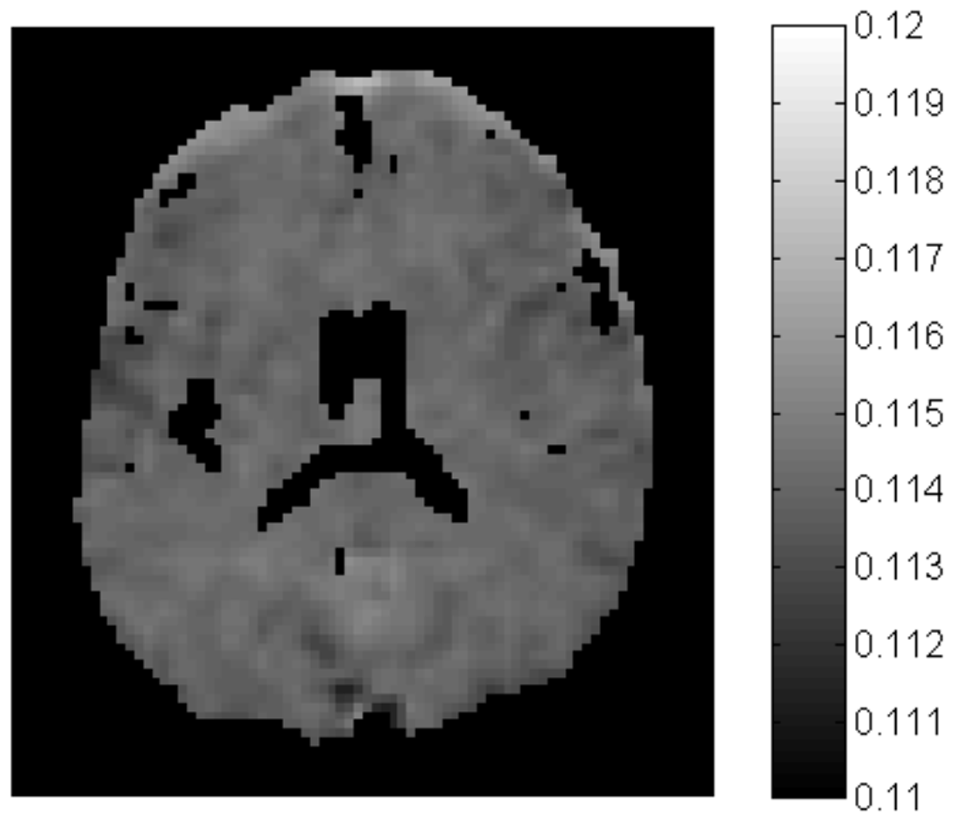
Power spectrum distribution in the slice shown in Fig. 3D. The intensity represents the ratio of the power (i.e., squared Fourier coefficient) for the frequency *f* = 0.01–0.1 Hz to the total power for all frequencies at *f*>0.

Power spectral analysis of the entire slice containing Fig. 1b shows that the power for the frequency range *f* = 0.01–0.1 Hz is ∼11–12% of the total power for all frequencies at *f*>0 across the brain parenchyma (Fig. 2). Further statistical analysis shows that the mean percent power of lower frequency (*f* = 0.01–0.1 Hz) in the WM is 11.41% with standard deviation of 0.03%. This mean percent power is slightly although significantly greater than that in the GM, which has a mean percent power of 11.39% with standard deviation of 0.04% in the low frequency range of *f* = 0.01–0.1 Hz (p<0.01).

The analyses of intensity profiles and power spectra above demonstrate that MRI signals across the parenchyma contain a similar magnitude of variations in the temporal domain, and that WM possesses a slightly greater power in low frequency fluctuations than GM. These observations indicate that similar to GM, WM also conveys information that may be similarly detectable using appropriate signal analysis.

### Spatio-temporal correlation of resting state BOLD signals

Anisotropic temporal correlations between resting state signals are observed in many WM regions in all the six subjects studied. Representative findings in the corpus callosum (CC) of a female subject (Subject One, age = 29 yrs) and those along the left optic radiation (OR) of a male subject (Subject Two, age = 25 yrs) are reported below.

**Figure 3:**
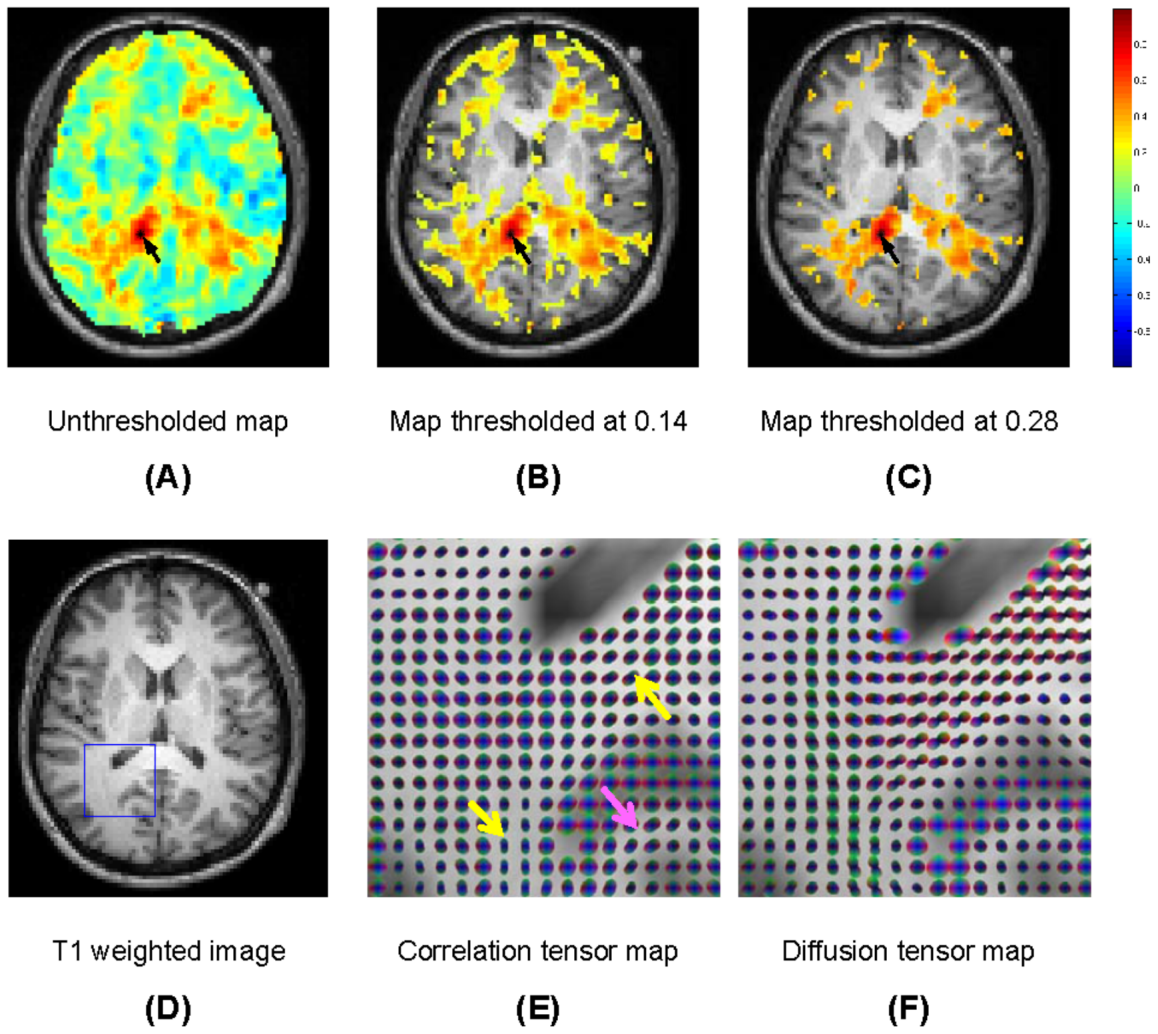
Maps of temporal correlations of BOLD signals to a seed in the corpus callosum. The background is the anatomic image. Shown from (A) to (C) are maps thresholded at different levels of temporal correlations (see text for explanations). Shown from (D) to (F) are respectively a slice of anatomic T1 weighted image, map of spatio-temporal correlation tensors and map of diffusion tensors for the region demarked in (D).

The spatial distributions of temporal correlations of resting state signals with a point seeded in the CC are shown in the top row of Fig. 3 for Subject One. From (A) to (C) are respectively the correlations of the entire slice with a seed indicated by the dark arrow, correlations thresholded at correlation coefficient *r*>0.14, and correlations thresholded at *r*>0.28. The threshold value of 0.14 is chosen on the basis of data from Subject One (described in the next sub-section) such that it is the lower bound of mean correlations along WM fiber tracts in the whole brain. The correlation map is further thresholded at 0.28 to remove many “false positive” voxels structurally unrelated to the CC. It can be seen from Fig. 3A–C that voxels in the CC of both hemispheres tend to show much higher temporal correlations to the seed than the vast majority of other WM voxels. These high correlations extend over long distances from the seed to reach the WM-GM interface, signifying there are synchronized variations in the CC.

**Figure 4:**
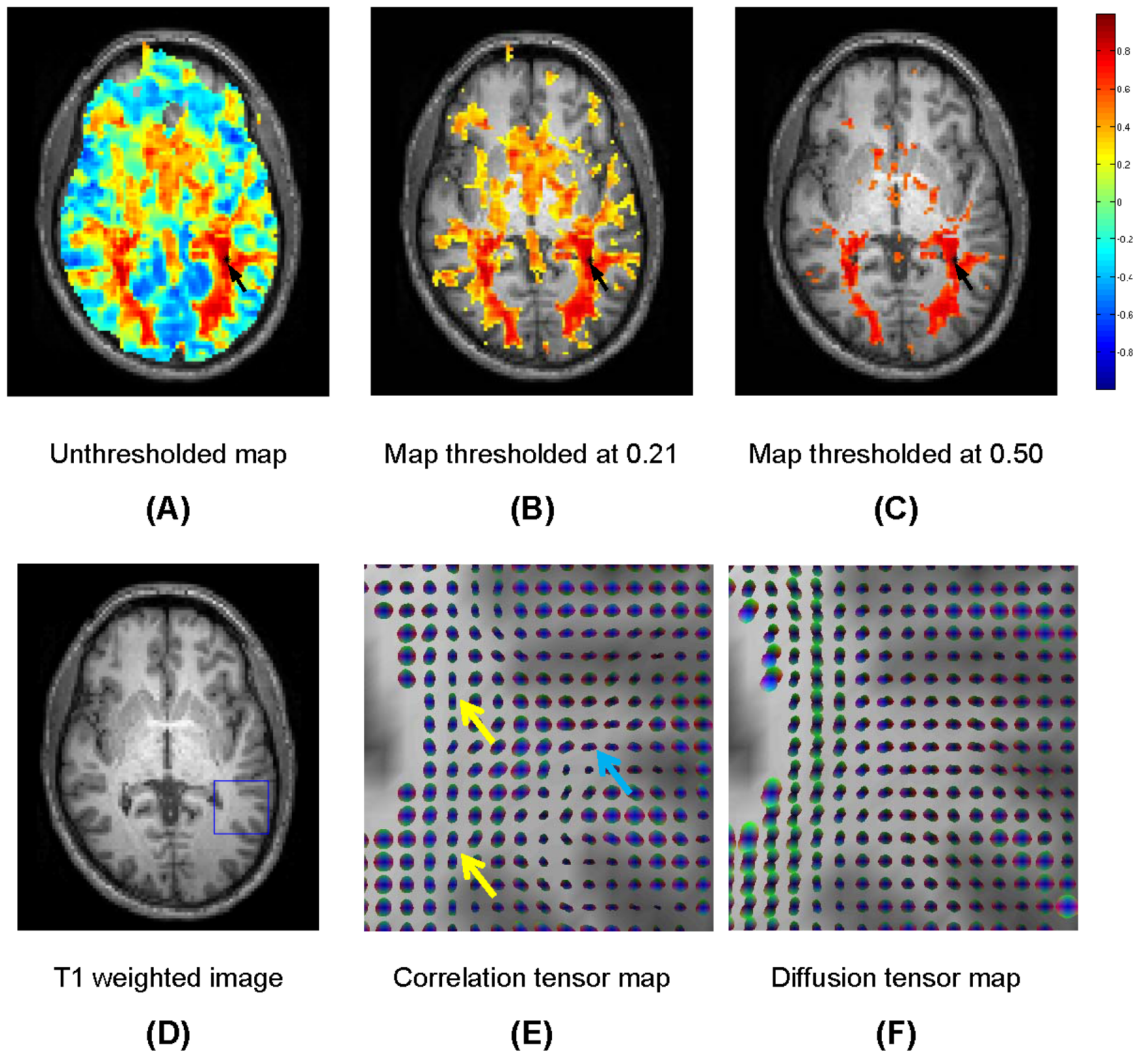
Maps of temporal correlations of BOLD signals to a seed in the left optic radiation. See captions for Fig. 3 for explanations of the individual images.

Maps of spatio-temporal correlation tensors and diffusion tensors computed for a region that contains a portion of the CC are shown in the bottom row of Fig. 3. In this figure (and in Fig.4 below as well), radiologic view conventions are used (image right  =  subject left), and the color scheme follows that adopted by the DTI community (red  =  left-right direction, green  =  anterior-posterior direction, blue  =  inferior-superior direction). The same portion of the T1 weighted image is shown underneath for anatomical reference. Voxels with very low time-averaged MRI signal intensities or mean diffusivity greater than 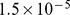 cm^2^/s were excluded.

The spatial-temporal correlation tensors in WM show clear patterns of anisotropy and suggest an underlying macroscopic structure. There is gross agreement in the patterns of the resting state correlation and diffusion tensor maps, particularly along some of the large WM tracts (pointed to by yellow arrows). Close inspection reveals that the correlation tensors sometimes tend to better delineate fine structures than the diffusion tensors, as evidenced by a clearer “U-turn” shaped pattern seen in the vicinity of the gray matter gyri (pointed to by the pink arrow).

The spatial distributions of temporal correlations of BOLD-sensitive signals with a point seeded in the left OR are shown in the top row of Fig. 4 for Subject Two. From (A) to (C) are respectively the correlations of the entire slice with a seed indicated by the dark arrow, correlations thresholded at *r*>0.21, and correlations thresholded at *r*>0.50. The threshold value of 0.21 is chosen similarly to that in Fig. 3(B) but on the basis of data from Subject Two. The threshold value of 0.50 is chosen to further remove many voxels structurally unrelated to the left or right OR. It can be seen that, similar to the CC, voxels in the OR of both hemispheres tend to have higher temporal correlations than the vast majority of other WM voxels. These high correlations virtually extend to the entire course of both ORs and reach the WM-GM interface, which again signifies there are synchronized variations in the WM structure, and they are specific and confined to the known anatomical extent of the OR.

Maps of spatio-temporal correlation tensors and diffusion tensors computed for a region that contains a portion of the left OR are shown in the bottom row of Fig. 4. Again the spatio-temporal correlation tensors show clear patterns of anisotropy along the OR (pointed to by yellow arrows), which are grossly consistent with those in the diffusion tensor map as well. Furthermore, there are clear patterns of correlational anisotropy around the OR (pointed to by cyan arrows), which also agree with those observed in the diffusion tensor map.

### Temporal correlations of resting state signals along WM tracts

**Figure 5:**
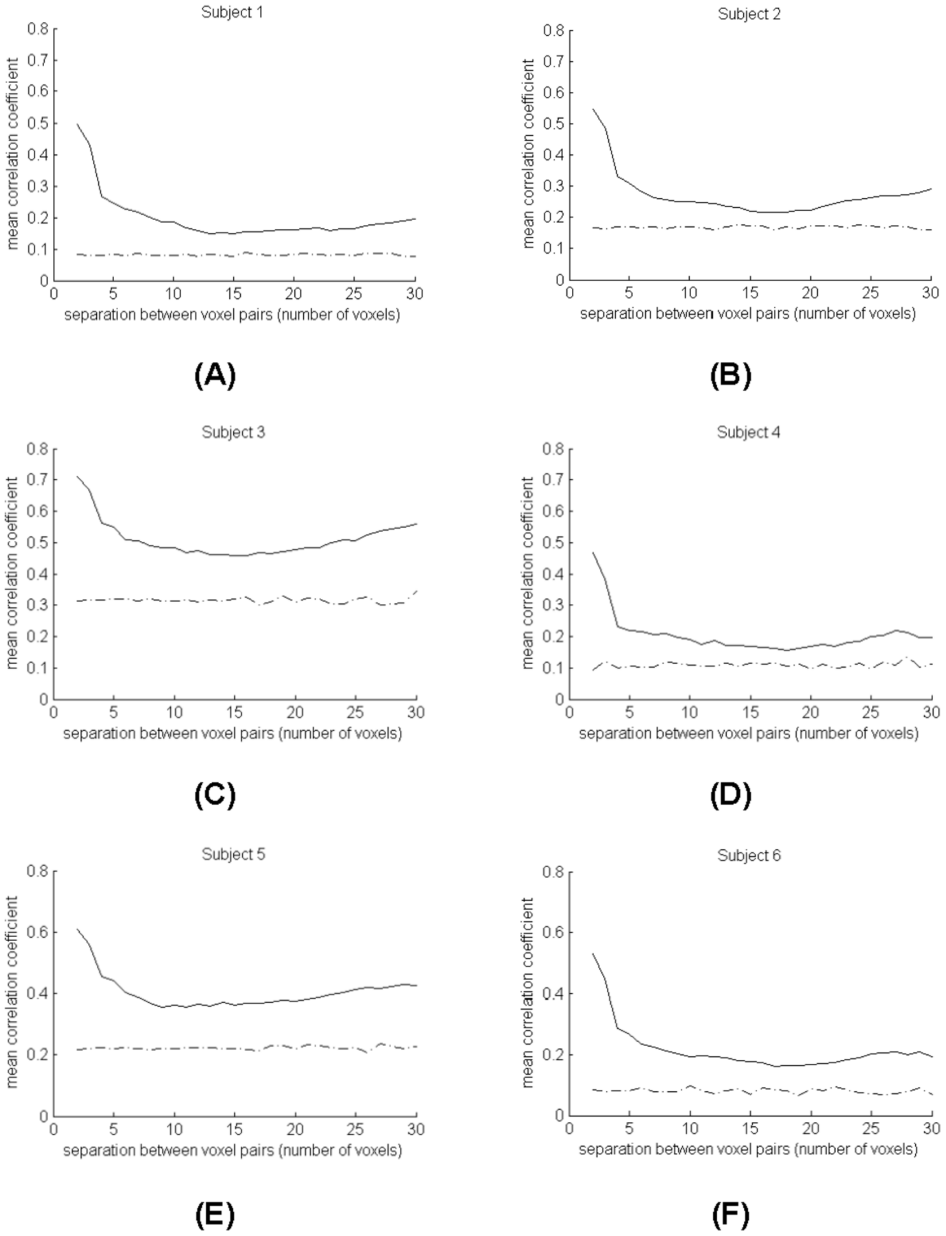
Mean correlation coefficients along fiber tracts versus fiber length. Each plot is for a different subject.

We evaluated the variation of temporal correlations in resting state data along individual WM tracts over increasing voxel separations. Variations of correlation coefficients of the MRI signals along the length of fibers tracked from DTI data of the six adult subjects studied are shown in Fig. 5. Voxel pairs along the same fiber tract were grouped according to the distance of their separation (i.e. fiber length), and their temporal correlation was measured as a function of that separation (from 4 mm to 60 mm apart). The mean correlation coefficient at each fiber length is shown as the solid curve, and the mean correlation coefficient of the same number of random voxel pairs in the WM is shown as the dash-dotted curve.

It can be seen that the mean correlation coefficient is higher at short fiber lengths and clearly persists over multiple voxel dimensions and is significantly higher than with randomly chosen voxels. Comparisons in the correlation coefficients between pairs of points along the same fiber and those selected at random show that the two groups are significantly different at each fiber length (p<0.05 at each distance), implying that the correlations along WM tracts are significantly different from background levels unrelated to connectivity.

**Figure 6:**
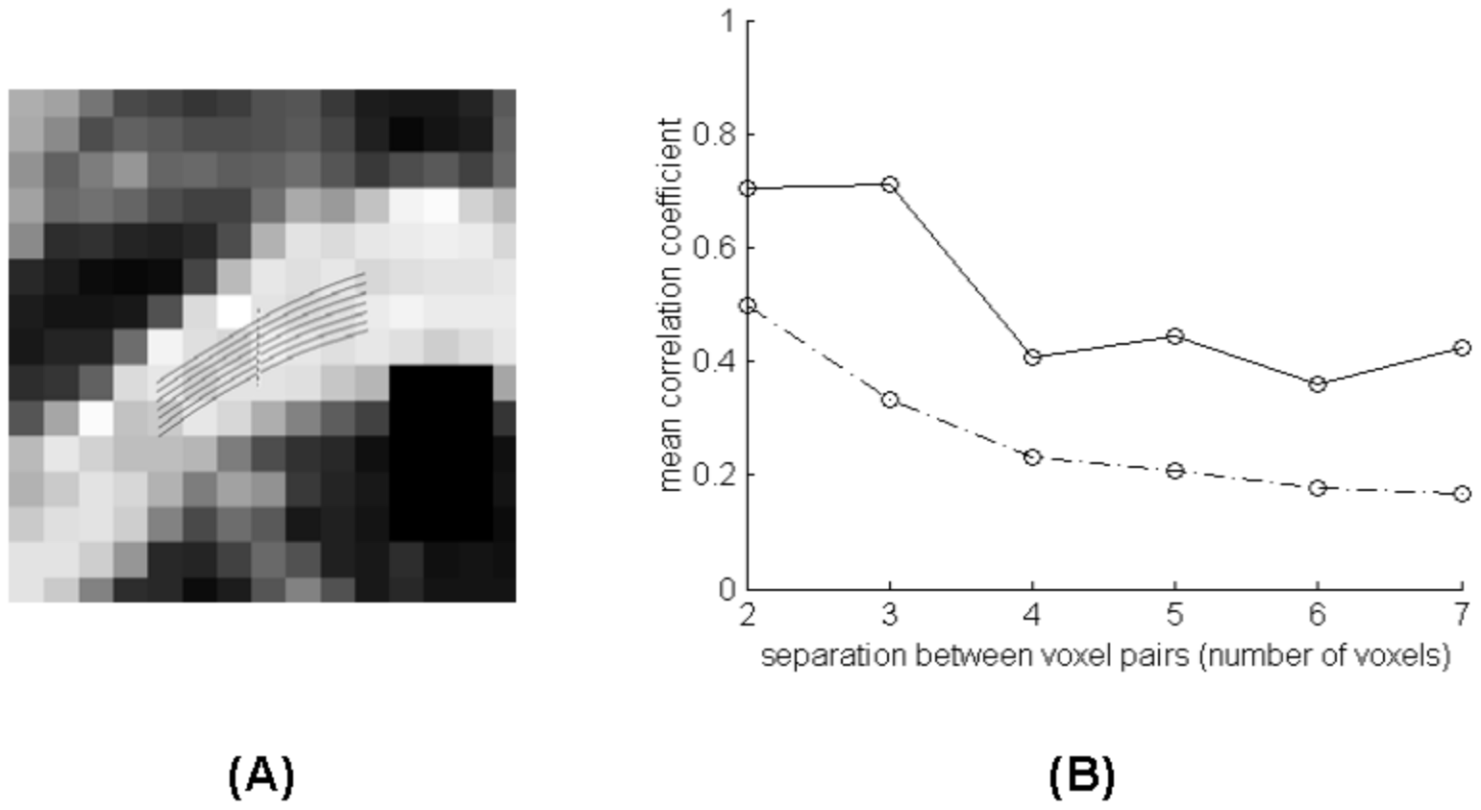
Detailed analysis of correlations along fiber segments tracked in the corpus callosum of Subject One. The dashed line at the left denotes the seed plane for fiber tracking. The solid curve at the right is the mean correlation coefficient over the seven segments and the dash-dotted curve is the mean correlation coefficient over 1000 random pairs of voxels in the white matter separated by the same distance.

Figure 6 shows a more detailed analysis of MRI signal correlations along seven fiber segments tracked in the CC of Subject One. Along the fiber length, the correlation coefficient for resting state signals averaged over the seven segments (solid curve) is significantly different from those averaged over 1000 random pairs of WM voxels separated by the same distance (dash-dotted curve), with p<0.05 at each distance.

### Functional pathways of optic radiation

**Figure 7:**
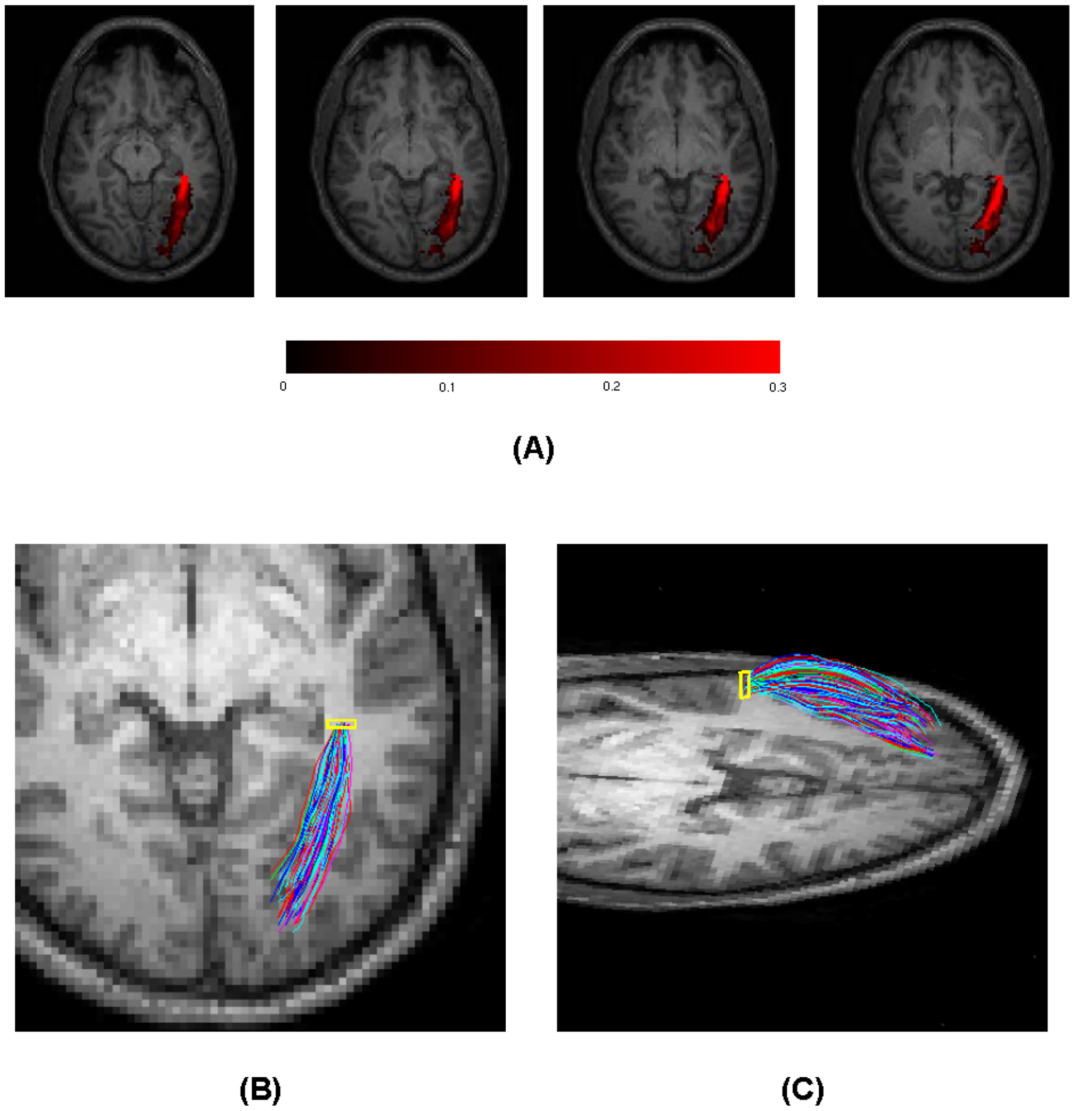
Reconstructed pathways of the left optic radiation of Subject Two. Top row: probability density maps of four selected slices that contain the left optic radiation, superimposed onto the corresponding T1 weighted image. Bottom row: randomly colored pathways back tracked from density and mean direction maps, rendered in an axial view (B) and oblique view (C). The yellow squares denote the seed region.

**Figure 8:**
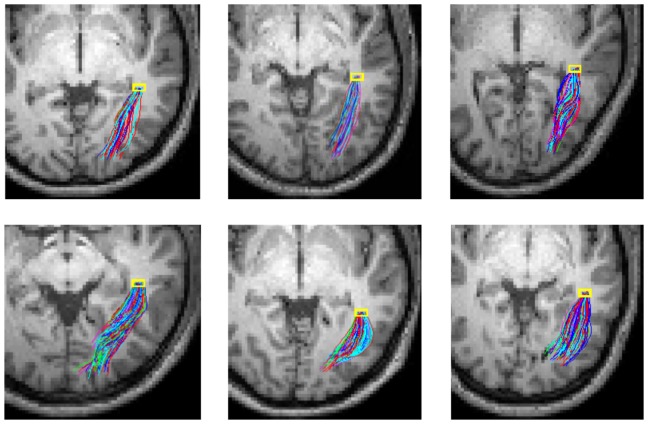
Reconstructed pathways of the left optic radiation of all the six subjects studied. The same set of parameters were used for probabilistic tracking in these subjects, and pathways are randomly colored and rendered in an axial view.

Probability density maps of the left OR in Subject Two based on resting state correlations and pathways reconstructed from these maps are shown in Fig. 7. Images in the top row (A) are density maps of four selected slices that contain the left OR, and the images in the bottom row are axial (B) and oblique (C) views of reconstructed pathways of the left OR. It can be appreciated that the density maps agree well with previous reports [18], [20], and pathways reconstructed are consistent with known functional anatomy [21]. Pathways of the left OR from all the six subjects studied are shown in Fig. 8. Although there are some variations across subjects, likely due to inter-subject anatomical variabilities, they all appear physiologically realistic. These demonstrate that the spatio-temporal correlation tensors derived from resting state BOLD signals have the potential of depicting functional structure of long range neural circuits.

## Discussion

Since the advent of the resting state fMRI paradigm, a variety of analysis methods have been proposed, including model-driven methods such as temporal correlation [5] and hierarchical clustering [22], or data-driven methods such as principal or independent component analyses [23], [24]. More advanced statistical techniques have also been proposed to infer “effective connectivity” between cortical regions by using dynamic causal modeling [25] or Granger causality analysis [26], but the inferences drawn therein remain controversial [27].

This study presents an alternative way of analyzing fMRI data, and demonstrates there are correlated signal fluctuations between voxels within both white matter and gray matter. Drawing upon temporal correlations of these BOLD-sensitive signals within neighboring voxels, this new approach measures the local anisotropy of these correlations, particularly in white matter, which appears to indicate underlying structure. Our resting state acquisitions indicate that there are synchronized signal variations within white matter, and quantitative analyses of these temporal correlations along fiber tracts defined by DTI demonstrate that the correlations are significantly higher than background correlations in white matter, and have similar temporal properties as BOLD fluctuations in gray matter. These provide the basis for the concept of using local spatio-temporal correlation tensors for visualization of white matter structure. The proposed analyses show gross similarities between correlation tensors and diffusion tensors in many white matter regions, and pathways reconstructed from spatio-temporal correlation maps appear plausibly realistic.

In principle, because axonal fibers are carriers of neural activity, the correlational anisotropy of BOLD-sensitive signals measured in white matter may indicate information propagation. Thus the local spatio-temporal correlation tensor derived from resting state data may characterize a local functional structure (i.e., a functionally active component of an anatomic structure defined by the diffusion tensor). In practice, the interpretation may be confounded by a number of processes that could degrade measured signals during imaging or post-processing procedures. These include the effect of point spread function during image formation and explicit or implicit smoothing during post-processing (such as spatial filtering or interpolation), all of which locally blur the signals and can create artificial correlations. We note, however, that the effect of point spread function by nature is isotropic, as evidenced by round tensors inside many gray matter regions in our experiments, and thus tends not to change the dominant direction of the correlation tensors and affect their interpretation. Typical smoothing also introduces an isotropic spreading effect unless anisotropic smoothing is specifically employed.

Potentially of higher impact are imaging artifacts typical of fMRI studies [28], which may complicate interpretations of observed correlational anisotropy. Major fMRI artifacts include head motion, signal drift due to instrument imperfection, and signal contaminations by cardiac and respiratory rhythmic modulations [29], [30]. To ameliorate these effects, conventional pre- and post-processing procedures were undertaken in this work. First, we minimized head motion by placing restricting pads within the head coil, corrected for head motion prior to data processing, and excluded subjects with movement greater than pre-defined thresholds. Second, we corrected for signal drift by using intensity normalization. Third, we reduced non-neuronal physiological noises by low pass filtering. We are aware that there are more sophisticated techniques for reducing these artifacts, perhaps with enhanced performance, such as removal of global signal drift with voxel-level linear modeling [31], and regressing out of cardiac and respiratory sources following independent component analysis [32]. These options can be considered in the future, along with more rigorous approaches to establishing appropriate false discovery rates. Note however that the residual effects of such artifacts would not explain the nature of the correlational maps shown in this study or be specific to particular white matter structures.

We recognize that, with a repetition time of 2 s, high frequency cardiac signals may be aliased into low frequencies. These aliased signals along with low frequency respiratory signals cannot be removed by low pass filtering. To assess the influence of these low frequency non-neuronal physiological signals, we constructed resting state correlation tensors with low pass filtering and RETROICOR [33], a common preprocessing method for reducing physiological noise. Quantitative comparisons of correlation tensors in a typical region that encompasses both gray matter and white matter show that low-pass filtering at 0.1 Hz had a major effect (∼16° in the tensor dominant direction) and correction with RETROICOR had a minor effect (<6° in the tensor dominant direction). This is consistent with other studies showing that high frequency noise has a significant effect on measurements of functional connectivity, in which contributions from non-neuro physiological noise including the cardiac and respiratory rhythms are relatively small [34]. We further found that, compared with low pass filtering alone, additional correction by RETROICOR had a minimal effect (∼3° in the tensor dominant direction), suggesting that low pass filtering has removed about half of the effects from cardiac and respiratory signals.

One of our major findings is that resting state MRI signals in brain white matter exhibit anisotropic correlations. We should point out that, in this work, two large white matter tracts are used for the purpose of proof-of-concept. In our additional experiments, many smaller white matter tracts are also visualized with the spatio-temporal correlation tensors, which show a good agreement with those delineated with diffusion tensors. Robust visualization of smaller white matter tracts however involves some preprocessing procedures for fMRI time series both in the frequency and spatial domains, which are under our active development. Nonetheless, the findings from this work, along with the recent report of BOLD effects in the corpus callosum [15], support the hypothesis that MRI signals reflecting neural activity might be reliably detected in white matter.

The mechanism underlying the generation of white matter MRI signal variations is however unclear. White matter consists of axons whose primary role is conducting action potentials that carry neural signals. The axonal fibers are in effect an “information highway” that conducts action potentials using the mechanism of saltatory propagation, which is achieved via electrical field propagation mediated by Ranvier nodes that are regularly spaced among axon segments [35]. It seems that white matter possesses high functional efficiency, which in turn requires much less oxygenated blood for energy supply than the gray matter where neuronal cell bodies reside, and thus may not be able to produce measurable BOLD signals. It is possible that the signal variations we report are not BOLD in origin, but instead reflect changes in M_0_ and/or T_2_ associated with non-vascular phenomena that affect the MR properties of axonal water. Continuing efforts are being made to further elucidate the contrast mechanism, and to characterize quantitatively structure-function relations on the basis of the spatio-temporal correlation tensors and diffusion tensors in the same volume.
